# SNP-based association study of kernel architecture in a worldwide collection of durum wheat germplasm

**DOI:** 10.1371/journal.pone.0229159

**Published:** 2020-02-14

**Authors:** Longqing Sun, Sisi Huang, Genlou Sun, Yujuan Zhang, Xin Hu, Eviatar Nevo, Junhua Peng, Dongfa Sun

**Affiliations:** 1 College of Plant Science and Technology, Huazhong Agricultural University, Wuhan, Hubei, China; 2 Biology Department, Saint Mary’s University, Halifax, Nova Scotia, Canada; 3 Institute of Evolution, University of Haifa, Mount Carmel, Haifa, Israel; 4 Germplasm Enhancement Department, Huazhi Biotech Institute, Changsa, Hunan, China; 5 Hubei Collaborative Innovation Center for Grain Industry, Jingzhou, Hubei, China; Murdoch University, AUSTRALIA

## Abstract

Durum wheat, genetic resource with favorable alleles is considered as natural gene pool for wheat breeding. Kernel size and weight are important factors affecting grain yield in crops. Here, association analysis was performed to dissect the genetic constitution of kernel-related traits in 150 lines collected from 46 countries and regions using a set of EST-derived and genome-wide SNP markers with five consecutive years of data. Total 109 significant associations for eight kernel-related traits were detected under a mix linear model, generating 54 unique SNP markers distributed on 13 of 14 chromosomes. Of which, 19 marker-trait associations were identified in two or more environments, including one stable and pleiotropic SNP *BE500291_5_A_37* on chromosome 5A correlated with six kernel traits. Although most of our SNP loci were overlapped with the previously known kernel weight QTLs, several novel loci for kernel traits in durum were reported. Correlation analysis implied that the moderate climatic variables during growth and development of durum are needed for the large grain size and high grain weight. Combined with our previous studies, we found that chromosome 5A might play an important role in durum growth and development.

## Introduction

Wheat is the most extensively grown commercial crop in the word [1]. The global demand for wheat is predicted to increase by 60% as the global population is estimated to be over nine billion by 2050 [2]. Therefore, genetic improvement of grain yield will still be the principal aim of wheat breeding. Considering the complex, polygenic inheritance, low heritability and significant influence of environment, yield improvement is faced with daunting challenges [3]. One of the important facets to achieve this goal is to explore novel genetic resources to discover genes that affect grain yield [4]. As a result, durum wheat (*Triticum turgidum* L. ssp. *Durum* Desf.) is often used as a bridge for transferring favorable alleles into bread wheat [5]. Wheat grain yield is a complex trait and determined by three main components, including spike number per unit area, kernel number per spike and kernel weight [6]. Kernel size, a key factor determining kernel weight and therefore grain yield [7], is also a quantitative trait with a complex genetic basis.

Comparative genomics approaches provide a powerful tool for gene discovery in wheat. Several QTLs and genes contributing to grain size and weight have gradually been isolated from wheat by using homology-based cloning of the orthologs in other cereal crops, including *TaGW2* [8], *TaSus2-2B* [9], *TaCwi-A1* [10], *TaCKX6-D1* [11], *TaGS-D1* [12], *TaGS5* [13], *TaTGW6-A1* [14], and *TaFlo2-A1* [15]. In recent years, more and more researchers have been attracted to study the functions of these genes. For instance, analysis of the function of *TaGW2* using CRISPR/Cas9 showed that mutation of *TaGW2* homoeologs resulted in the decrease of grain weight by affecting the grain size in bread wheat [16], which was in accordance with down-regulation of all the three homoeologs of *TaGW2* by RNA interference [17]. Haplotype *TaTGW6-A1a* associated with high grain weight was observed in approximately 80% of cultivars, indicating that it was a positively selected allele in wheat breeding [14]. *TaGS5-A1* haplotype was positively associated with high thousand-kernel weight in Chinese modern wheat [18]. In general, these results have helped us to understand the mechanism for kernel development in wheat.

High-density genetic linkage maps had been constructed to detect qualitative and quantitative trait loci for identifying candidate genes of many important traits in many species. A stable QTL *qKW-6A* was detected in both RIL population and DH population, suggesting that *qKW-6A* plays an important role in kernel width of wheat [19]. *TaTGW-7A*, a major QTL explaining 21.7*–*27.1% of phenotypic variance for thousand-kernel weight contributed significantly to wheat grain yield [20]. A single nucleotide polymorphism (SNP) in the promoter region of *1-FEH w3* gene was identified to be associated with thousand-kernel weight under drought conditions [21]. Six stable QTL were identified for controlling kernel size and weight in a recombinant inbred line population (RIL) [22]. *qKnps-4A*, a major stable QTL for kernel number per spike was identified by using the Affymetrix Wheat-660K single-nucleotide polymorphism (SNP) array [23].

However, linkage mapping has limitations including the basic requirement to create a bi-parental population segregating for target traits [24]. Another approach for identifying loci of traits is to employ association analysis with a large germplasm resources, known as linkage disequilibrium (LD) mapping, association mapping or genome-wide association studies (GWAS) [25], based on linkage disequilibrium (LD) or the non-independence of alleles in a natural population [26]. Association mapping has been proven to be successful in identifying marker-trait associations in plant [27]. Recent study has identified 26 quantitative trait loci (QTL) for kernel width and 27 QTL for kernel length in a historical United States wheat population [28]. A comprehensive genome-wide analysis using microsatellite markers and 90K iSELECT array identified *TaGW-6A* underlying thousand grain weight in a panel of European winter wheat varieties [29]. Twenty-seven markers were found to be associated with grain weight in a set of 230 elite Indian bread wheat cultivars [30]. Association analysis of 231 synthetic hexaploid wheats revealed that the loci associated with grain morphology were mainly distributed on homoeologous group 2, 3, 6 and 7 chromosomes [31]. Based on GBS markers, 17 grain size-associated SNPs were found in wild wheat *Aegilops tauschii* [32].

In common wheat, GWAS approach has been successfully employed to identify numerous candidate genes controlling a series of traits. Nevertheless, limited studies have utilized GWAS in durum wheat to dissect the genetic basis controlling kernel size and weight. In this study, we analyzed architecture of kernel characters in a panel of 150 durum lines collected from 46 countries and regions. As the result, a number of candidate genes were identified, which provides a useful resource for further functional studies to understand the molecular mechanism underlying grain development.

## Materials and methods

### Plant materials and field trials

One hundred fifty durum wheat accessions, consisting of 51 landraces and 99 cultivars from 46 countries and regions around the world, were used for association analysis in the study. This set of durum wheat was classified into 11 groups based on their geographic origins: East Asia (15), Central Asia (2), South Asia (6), Middle East (32), North America (33), Latin America (12), Oceania (7), Western Europe (14), Eastern Europe (5), South Africa (4), and North Africa (12). Details information was given in previous study [33]. All the accessions were cultivated in the experimental plot of Huazhong Agricultural University, Wuhan, Hubei of China (N30°32′ and E114°20′) in five consecutive years. During the 2013/2014, 2014/2015, 2015/2016 2016/2017 and 2017/2018 cropping seasons, the durum wheat accessions were planted in late October of first year and harvested in June of next year for each cropping season. Each accession was sown in four rows with 1 m in length and 20 cm between rows, 8 plants in each row. The experimental field belongs to the type of heavy loam with PH value of about 6.2. Water was sprayed evenly after sowing by sprinkling irrigation system. The soil moisture for durum seedling was about 70% of field water capacity. Compound fertilizer (825 kg/ha) was used as base fertilizer and 150 kg/ha of urea fertilizer was used as top dressing. Each field trial was conducted in a randomized complete block design with three replications.

### Phenotypic evaluation

The kernel traits were measured at maturity. Thirty spikes from the individual plant of each line were randomly collected from the middle row in each plot and sundried. Then, all spikes from three different field experimental trials were mixed together for threshing. About 300 fully filled seeds of every line were randomly selected to obtain kernel parameters using SC-G phenotyping system (Wanshen Detection Technology Co., Ltd., China) [34]. In total, 8 traits were measured or calculated: kernel area (KA), kernel circumference (KC), kernel diameter (KD), kernel length (KL), kernel roundness (KR), kernel width (KW), length/width ratio (L/W), thousand kernel weight (TKW).

### Phenotypic data and correlation analysis

Descriptive statistical analysis and analysis of variance (ANOVA) of phenotypic data, broad-sense heritability (*H*^*2*^) for each trait, and Pearson correlation coefficients analysis among different traits were calculated by using SPSS 21.0 (https://www.ibm.com/support/pages/node/213045). Kolmogorov-Smirnov test was performed to test normal distribution of each trait. Origin Pro2017 (http://www.chem.ox.ac.uk/origin/) was used to draw figures of frequency distribution for the examined traits. In order to calculate the mean values of each trait, the best linear unbiased prediction (BLUP) method was estimated using a mixed-effects model implemented in the lme4 package [35]. Correlation analysis between eight evaluated kernel traits and three climate factors was performed by using SPSS 21.0. The critical developmental stages for durum wheat after overwintering were divided into three important growth stages (I, II, and III). Stage I represented the growth period of regreening for durum around the time in February, and stage II corresponded with the growth of jointing stage in March. Due to the growth rate vary with different lines of the durum population at the heading and flowering, grain filling, and ripening phases, stage III was the combination period from heading to ripening (April-June period). The average values of temperature, sunlight and rainfall precipitation for each of the three stages, which were collected from weather station in Hubei province from 2014 to 2018, were set as the climate parameters. Correlation coefficients of kernel phenotypes with climate factors were based on the mean values of the kernel traits and climate parameters.

### Association analysis

SNP genotyping was performed on Illumina Bead Array platform and Golden Gate Assay (Illumina, San Diego,CA) at the Genome Center of the UC Davis according to the manufacturer’s protocols. The SNP markers used in this study were developed from the EST database. After the process of quality management, 1366 single nucleotide polymorphisms (SNP) markers covering the whole genome of durum were used to genotype the durum accessions. The rate of change in the Napierian logarithm probability relative to standard deviation (ΔK) suggested that the best structure was K = 2. More details were described in previous study [33]. The mean marker density of 95–96 markers per chromosome, ranging from 66 (3B) to 130 (7A) for all the 14 chromosomes, were used to calculate the extent of LD. At the chromosomal level, the LD decay distance ranged from 1.90 Mb (1A) to 96.05 Mb (2A) [33, 36]. The associations were estimated under the mixed linear model (MLM) using software TASSEL 3.0 (http//www.Misogynistic.net/tassel), accounting for Q-Matrix of the population structure as a covariate and pair-wise kinship coefficients (K matrix) as random effects [33]. Significance of associations between markers and traits was evaluated by *P*-value, and the QTL effects were estimated using marker-R^2^. *P* = 0.01 was used to declare the significant association signals according to our previous study [36].

### The Physical Position Identification and Candidate Gene Prediction

The EST sequence of each significantly associated SNP marker was analyzed using translated nucleotide BLAST software from NCBI (http://www.ncbi.nlm.nih.gov/) for candidate genes prediction. Their functions were predicted based on the level of homology identity with the other species. The durum genome was downloaded from the International Durum Wheat Genome Sequencing Consortium (*Triticum turgidum* Durum Wheat Svevo, RefSeq Rel. 1.0). To obtain the physical positions of the our SNP sequences, and search previously identified QTLs which overlapped with our markers in durum, all EST sequences of the significant SNP markers were analyzed using Nucleotide BLAST to gain information in durum reference genome (https://wheat.pw.usda.gov/GG3/jbrowse_Durum_Svevo).

## Results

### Phenotypic Variation

**Fig 1:**
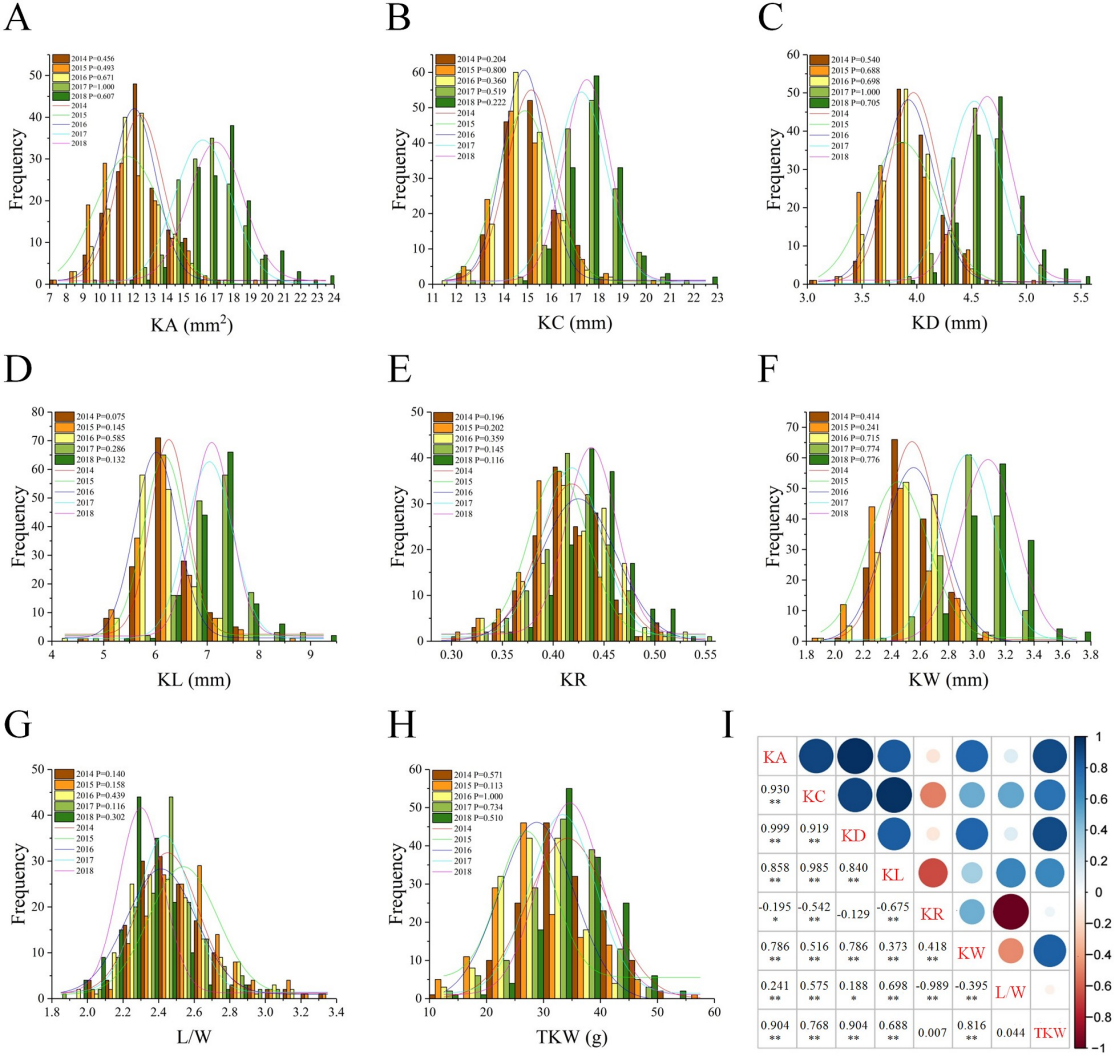
Frequency distribution of kernel-related traits. **(A)** kernel area (KA), **(B)** kernel circumference (KC), **(C)** kernel diameter (KD), **(D)** kernel length (KL), **(E)** kernel roundness (KR), **(F)** kernel width (KW), **(G)** length/width ratio (L/W), **(H)** thousand kernel weight (TKW) for the durum natural population in five consecutive years. P value of Kolmogorov-Smirnov test for each trait in each year was shown. Normal distribution could be accepted if P > 0.05, and the expected normal distributions were represented by the trend lines. **(I)** Correlation coefficients among these eight evaluated kernel traits were calculated by using their five-years best linear unbiased prediction (BLUP) values. The two-tailed *t* test was applied to test the significance of correlation coefficients (*P < 0.05, ** P < 0.01).

In total, eight kernel-related traits were measured: kernel area (KA), kernel circumference (KC), kernel diameter (KD), kernel length (KL), kernel roundness (KR), kernel width (KW), length/width ratio (L/W), thousand kernel weight (TKW). The frequency distribution of these traits was showed in Fig 1. A large range of variation for each investigated trait was detected in this natural population. Moreover, the trait distribution pattern was similar among five years for most traits (Fig 1A–1H). Therefore, the kernel traits showing the typical quantitative in heritance were used for association mapping analysis. The phenotypic values for each of the six kernel-related traits (KA, KC, KD, KL and KW) in the years from 2014 to 2016 were all lower than those in the years from 2017 to 2018 (S1 Fig). The descriptive statistics of the investigated traits for the population were shown in Table 1.

**Table 1:** Descriptive statistics of phenotypic performance and broad-sense heritability for the evaluated kernel traits.

Trait^a^	Year	Mean	Range		SD^b^	CV (%)^c^	*H*^*2*^ (%)^d^
			Minimum	Maximum			
KA (mm^2^)	2014	12.54	7.61	17.16	1.60	12.76	62.43
	2015	12.01	7.84	19.90	1.93	16.07	
	2016	12.19	8.05	17.38	1.58	12.96	
	2017	16.14	9.96	21.31	1.84	11.40	
	2018	17.15	11.96	23.47	2.01	11.72	
KC (mm)	2014	15.28	12.21	18.39	1.15	7.53	76.40
	2015	15.05	12.03	20.75	1.29	8.57	
	2016	15.00	11.40	18.87	1.11	7.40	
	2017	17.32	12.72	21.05	1.20	6.93	
	2018	17.59	14.03	22.14	1.19	6.77	
KD (mm)	2014	3.98	3.10	4.67	0.25	6.28	59.88
	2015	3.89	3.15	5.02	0.31	7.97	
	2016	3.92	3.23	4.69	0.25	6.38	
	2017	4.52	3.70	5.19	0.25	5.53	
	2018	4.66	3.07	5.45	0.26	5.58	
KL (mm)	2014	6.30	4.86	7.88	0.53	8.41	80.91
	2015	6.22	4.74	8.78	0.58	9.32	
	2016	6.13	4.45	7.94	0.51	8.32	
	2017	7.05	4.93	8.92	0.56	7.94	
	2018	7.08	5.43	9.20	0.53	7.49	
KR	2014	0.41	0.31	0.51	0.04	9.76	74.14
	2015	0.40	0.30	0.51	0.04	10.00	
	2016	0.42	0.32	0.53	0.04	9.52	
	2017	0.41	0.32	0.54	0.03	7.32	
	2018	0.43	0.34	0.53	0.03	6.98	
KW (mm)	2014	2.57	2.06	3.09	0.18	7.00	50.50
	2015	2.48	1.95	3.09	0.23	9.27	
	2016	2.54	1.93	3.13	0.20	7.87	
	2017	2.92	2.33	3.36	0.19	6.51	
	2018	3.08	2.60	3.75	0.20	6.49	
L/W	2014	2.49	2.01	3.34	0.23	9.24	73.68
	2015	2.57	2.02	3.36	0.23	8.95	
	2016	2.46	1.94	3.12	0.23	9.35	
	2017	2.45	1.87	3.08	0.20	8.16	
	2018	2.32	1.98	2.88	0.17	7.33	
TKW (g)	2014	35.03	13.76	52.43	6.95	19.85	40.52
	2015	29.60	11.15	56.66	8.42	28.45	
	2016	28.66	11.28	44.85	6.30	22.00	
	2017	32.43	12.17	47.74	6.68	20.59	
	2018	35.17	14.44	52.85	6.18	17.58	

^a^*KA* kernel area, *KC* kernel circumference, *KD* kernel diameter, *KL* kernel length, *KR* kernel roundness, *KW* kernel width, *L/W* length/width ratio, *TKW* thousand kernel weight.

^b^SD standard deviation.

^c^CV coefficient of variation.

^d^*H*^*2*^ broad-sense heritability.

The coefficients of variation (CV) among genotypes for all the phenotypic traits in each environment ranged from 5.53 to 28.45%. However, the variation of each trait was different among years. For instance, the variation for the KA ranged from 7.61 to 17.16 mm (mean ± *SD* = 12.54 ± 1.60 mm) in year 2014, but from 11.96 to 23.47 mm (17.15 ± 2.01 mm) in 2018. TKW had the highest CV among these traits, whereas KD had the lowest CV (Table 1). Most of the traits have high broad-sense heritability (*H*^*2*^ > 60%), indicating that a large portion of phenotypic variance for kernel traits were stable and mainly contributed by genotypic effects.

**Fig 2:**
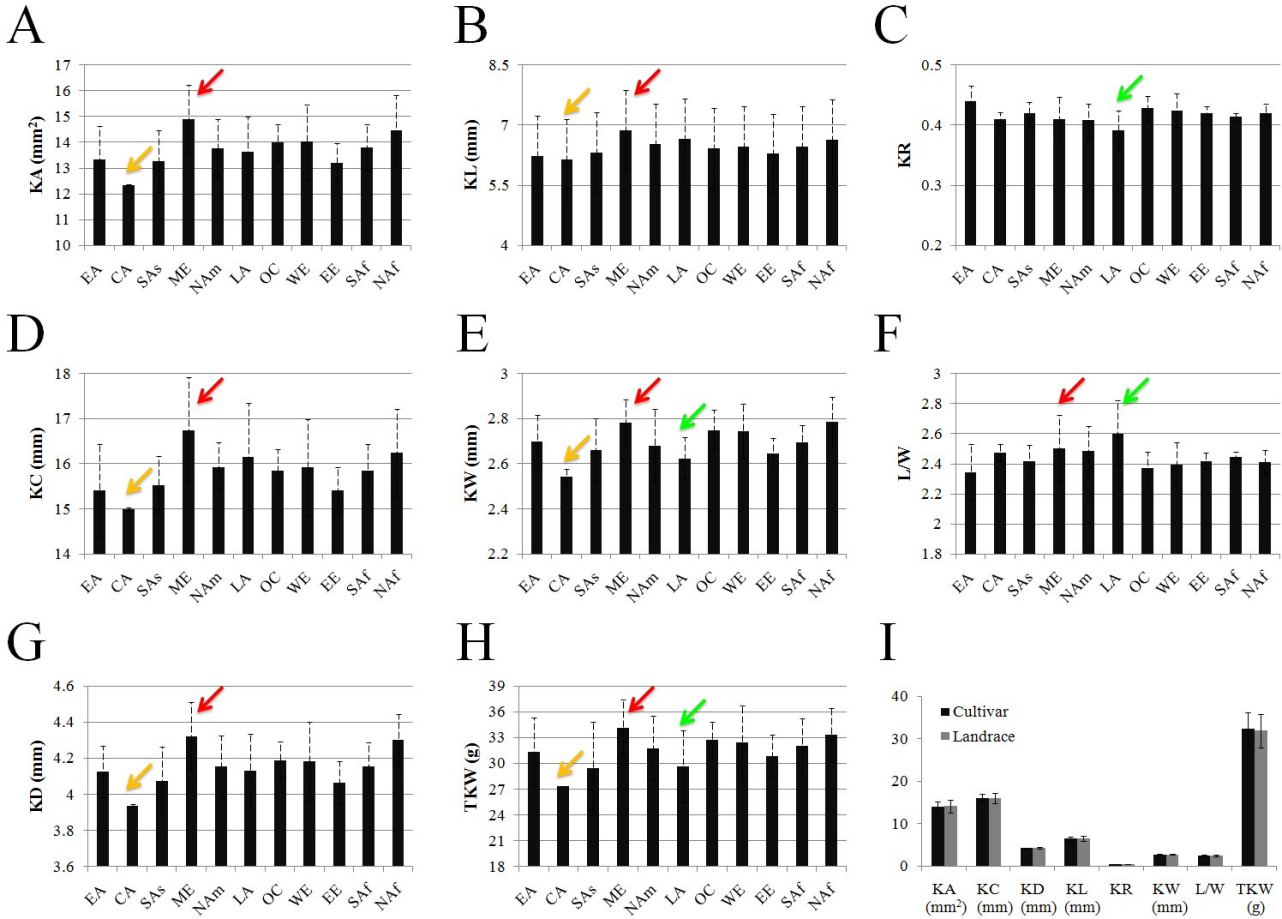
Phenotypic variation of measured traits in durum wheat from different regions. The set of durum wheat was classified into 11 groups based on their geographic origins: EA, East Asia (15); CA, Central Asia (2); SAs, South Asia (6); ME, Middle East (32); NAm, North America (33); LA, Latin America (12);OC, Oceania (7); WE, Western Europe (14); EE, Eastern Europe (5); SAf, South Africa (4); NAf, North Africa (12). Red arrow represented the values of kernel traits in Middle East. Green arrow represented the values of kernel traits in Latin America. Yellow arrow represented the values of kernel traits in Central Asia. **(A)** KA, **(B)** KL, **(C)** KR, **(D)** KC, **(E)** KW, **(F)** L/W, **(G)** KD, **(H)** TKW, **(I)** Comparison of each kernel-related trait between landraces and cultivars.

The 150 durum accessions were divided into 11 geography of origins based on their sources and locations [33]. The potential relationship between kernel traits and geographical regions was estimated to explore the effect of regional characteristics on phenotypic traits. Phenotypic variability varied from the different regions (Fig 2). The values of KA, KC, KD, KL, KW, L/W and TKW for durum accessions from Middle East were almost all higher than those in the other 10 geographical regions (Fig 2A, 2B and 2D–2H). The values of KR, KW and TKW for durum wheat from Latin America were lower than those in other regions except Central Asia (Fig 2C, 2E and 2H), but the value of L/W of durum germplasm in Latin America was the highest (Fig 2F). The values of KA, KC, KD, KL, KW, and TKW for durum from Central Asia were the lowest (Fig 2A, 2B, 2D, 2E, 2G and 2H). However, it is unlikely to be typical case due to only two durum wheat accessions from this region included in this study.

Comparison between landraces and cultivars did not show significant rule and trend of the kernel-related traits (Fig 2I), and significant differences for KA, KC, KD, KL, KR, KW, L/W and TKW between landraces and cultivars of durum (S2 Fig).

### Correlation among the observed traits

Correlation analysis was performed among eight evaluated kernel traits. Out of the 28 possible correlation pairs, there were 23 highly significant (p< 0.01) and two significant (p< 0.05) correlations. Moreover, as shown in Fig 1I, the correlations between phenotypic traits were relatively high, most of them achieved over 80%. Highly positive correlations were observed among KA, KC, KD and KL (r = 0.840–0.999). KA, KC, KL and L/W showed significantly negative correlations with KR, while KR was significantly positive correlated with KW. KW showed significantly positive correlations with other kernel traits except for significantly negative correlations with L/W. KA, KC and KL showed significantly positive correlations with L/W, while KR and KW showed significantly negative correlations with L/W (Fig 1I). The correlation between KR and KD was very low, which indicated that the genetic determinant of these two parameters were relatively independent. KR had significantly negative correlation with KA, which suggested tradeoffs between them. Interestingly, TKW was positively correlated with five kernel traits, KA, KC, KD, KL and KW. However, no significant correlation was found for TKW with either KR or L/W (Fig 1I).

### Associations for kernel-related traits

In our study, 1366 single nucleotide polymorphisms (SNP) markers covering the whole genome of durum were used to genotype 150 durum germplasm accessions. Details on the population structure and the linkage decay were described in our previous study [33]. Here, association analyses were performed on the 8 kernel traits and SNP markers. In total, 109 trait-marker associations (MTAs) were identified by MLM for the all kernel traits across five consecutive years. It was found that the numbers of MTAs in the year 2014, 2017 and 2018 were similar (S1 Table). The complete list of MTAs was shown in Table 2. The number of SNPs detected for a trait varied among the years. A total of 17 SNP markers were detected for KR in year 2016, which was the maximum amount of SNPs in single year for single trait (S3A Fig). About 94.5% of the significant SNPs for kernel traits exhibited marker-R^2^ < 10%, only a few SNPs associated with KA, KC, KL, KR and L/W showed R^2^ ≥10% (S3B Fig). The results implied that the kernel-related traits in durum are mainly controlled by many loci with minor effect. As the most reliable method in detecting significant associations, BLUP was calculated to minimize the errors caused by simple means of all the years [37]. Only 11 significant MTAs for evaluated traits of kernel were obtained based on BLUP values, and no significant associations were found for KW (S2 Table). No SNP with R^2^ >10% was detected for all measured traits under BLUP model (S3C Fig).

**Table 2:** Summary of SNPs significantly associated with kernel traits across five years.

Trait	Marker	Allele	Environment	P value	R^2^
KA	BE500291_5_A_37	T/C	2015	0.0003	0.1100
			2016	0.0016	0.0702
			2017	0.0094	0.0471
			2018	0.0043	0.0568
			Blup	0.0016	0.0703
	BE445667_6_B_Y_285	A/C	2016	0.0095	0.0469
	CD452967_5_B_Y_229	T/C	2017	0.0058	0.0534
KC	BE500291_5_A_37	T/C	2015	0.0000	0.1250
			2016	0.0006	0.0839
			2017	0.0039	0.0585
			2018	0.0008	0.0789
			Blup	0.0005	0.0871
	BF483039_7_A_Y_202	A/G	2017	0.0058	0.0723
KD	BE500291_5_A_37	T/C	2015	0.0004	0.0899
			2016	0.0030	0.0630
			2018	0.0060	0.0535
			Blup	0.0023	0.0665
	CD453605_6_B_427	A/G	2016	0.0088	0.0678
			2018	0.0060	0.0732
	BF292193_7_B_N_78	A/C	2014	0.0086	0.0492
	CD452967_5_B_Y_229	T/C	2017	0.0008	0.0816
KL	BE500291_5_A_37	T/C	2015	0.0000	0.1265
			2016	0.0007	0.0812
			2017	0.0039	0.0583
			2018	0.0004	0.0893
			Blup	0.0004	0.0896
	BQ168780_5_B_995	C/G	2014	0.0025	0.0847
			2015	0.0065	0.0709
	BF483039_7_A_Y_202	A/G	2017	0.0070	0.0697
	BG274985_5_A_Y_267	T/C	2018	0.0096	0.0467
	BE403211_5_A_Y_601	A/G	2014	0.0044	0.0930
KR	BF474023_3_A_Y_425	T/C	2014	0.0043	0.0577
			2015	0.0009	0.0780
			2016	0.0036	0.0605
			2017	0.0079	0.0501
			Blup	0.0027	0.0642
	BE404377_4_B_Y_333	T/C	2015	0.0070	0.0701
			2016	0.0059	0.0733
			2017	0.0078	0.0692
	BF474862_5_A_762	T/C	2014	0.0071	0.0861
			2016	0.0017	0.1094
			Blup	0.0032	0.0992
	BE352626_4_A_Y_110	T/C	2014	0.0091	0.0480
			2015	0.0061	0.0529
	BM140538_2_B_133	A/G	2014	0.0061	0.0530
			2016	0.0096	0.0475
	BE404912_6_B_Y_488	T/G	2017	0.0009	0.1201
	BE405604_2_A_Y_353	A/T	2015	0.0065	0.0522
	BE425301_4_A_Y_160	A/G	2016	0.0058	0.0541
	BE446480_2_A_N_24	A/G	2015	0.0093	0.0475
	BE488358_2_B_N_620	T/G	2015	0.0085	0.0487
	BE517914_3_A_Y_81	T/G	2015	0.0023	0.0866
	BE591739_4_A_Y_131	T/C	2015	0.0065	0.0520
	BF482356_4_B_Y_504	A/C	2015	0.0095	0.0658
	BG262421_6_A_87	A/G	2015	0.0073	0.0695
	BG263233_1_A_Y_836	A/G	2016	0.0074	0.0508
	BG605368_2_A_156	T/C	2016	0.0036	0.0976
	BQ161779_6_B_Y_185	C/G	2016	0.0074	0.0508
	CD452413_3_B_Y_189	T/C	2014	0.0095	0.0816
KW	BF291774_6_B_519	A/G	2014	0.0057	0.0733
	BF292193_7_B_N_78	A/C	2014	0.0072	0.0509
	BE497375_7_A_Y_191	A/G	2016	0.0033	0.0617
	CD452967_5_B_Y_229	T/C	2017	0.0006	0.0833
	BE637838_7_A_Y_208	A/G	2018	0.0054	0.0739
	BE499652_7_A_Y_391	A/G	2018	0.0058	0.0727
	BE495175_3_B_Y_443	A/T	2018	0.0060	0.0723
	BE517872_2_A_N_504	A/G	2018	0.0064	0.0712
	BE498763_6_A_Y_318	A/G	2018	0.0064	0.0712
	BE499248_7_B_Y_63	A/T	2018	0.0070	0.0857
	BG604507_4_B_383	T/C	2018	0.0071	0.0856
L/W	BF474023_3_A_Y_425	T/C	2014	0.0048	0.0559
			2015	0.0007	0.0815
			2016	0.0020	0.0672
			2017	0.0038	0.0589
			2018	0.0040	0.0580
			Blup	0.0015	0.0715
	BM140538_2_B_133	A/G	2014	0.0086	0.0482
			2016	0.0096	0.0469
			2017	0.0095	0.0470
	BF474862_5_A_762	T/C	2016	0.0021	0.1047
			2017	0.0096	0.0806
			Blup	0.0069	0.0858
	BF292264_7_A_779	C/G	2014	0.0088	0.0665
			2015	0.0060	0.0714
			Blup	0.0081	0.0677
	BE517914_3_A_Y_81	T/G	2015	0.0012	0.0949
			Blup	0.0064	0.0711
	BE500291_5_A_37	T/C	2014	0.0016	0.0704
			2018	0.0054	0.0541
	BE352626_4_A_Y_110	T/C	2014	0.0072	0.0506
			2015	0.0043	0.0566
	CD452413_3_B_Y_189	T/C	2014	0.0068	0.0861
	BQ161779_6_B_Y_185	C/G	2016	0.0028	0.0627
	BG263233_1_A_Y_836	A/G	2016	0.0028	0.0627
	BF483091_6_A_357	T/G	2016	0.0080	0.0492
	BE604119_6_B_733	T/C	2016	0.0087	0.0668
	BE498892_2_A_208	T/C	2014	0.0078	0.0683
	BE497375_7_A_Y_191	A/G	2016	0.0038	0.0587
	BE496986_6_A_110	T/C	2016	0.0060	0.0722
	BE495116_4_A_Y_239	T/C	2016	0.0080	0.0492
	BE446087_3_B_Y_750	A/T	2016	0.0080	0.0492
	BE445508_3_B_Y_209	A/G	2016	0.0080	0.0492
	BE443253_4_B_Y_414	A/T	2015	0.0073	0.0686
	BE443010_7_B_354	A/G	2016	0.0041	0.0776
	BE425301_4_A_Y_160	A/G	2016	0.0062	0.0525
	BE406351_2_B_Y_100	C/G	2016	0.0080	0.0492
	BE404977_4_B_Y_227	T/G	2018	0.0031	0.0612
	BE404912_6_B_Y_488	T/G	2017	0.0078	0.0839
	BE404377_4_B_Y_333	T/C	2016	0.0025	0.0852
	BE404332_2_B_29	T/G	2018	0.0066	0.0515
	BE403597_2_B_Y_552	T/C	2016	0.0080	0.0492
TKW	BE500291_5_A_37	T/C	2015	0.0029	0.0625
			2018	0.0071	0.0507
			Blup	0.0090	0.0476
	BF483039_7_A_Y_202	A/G	2014	0.0048	0.0753
	BE405269_4_B_84	A/C	2015	0.0093	0.0472
	BE425919_3_A_592	A/T	2016	0.0036	0.0595
	BE442666_4_B_Y_327	T/C	2017	0.0083	0.0673
	CD452967_5_B_Y_229	T/C	2017	0.0015	0.0713
	BE636872_6_A_119	A/G	2018	0.0083	0.0828

**Fig 3:**
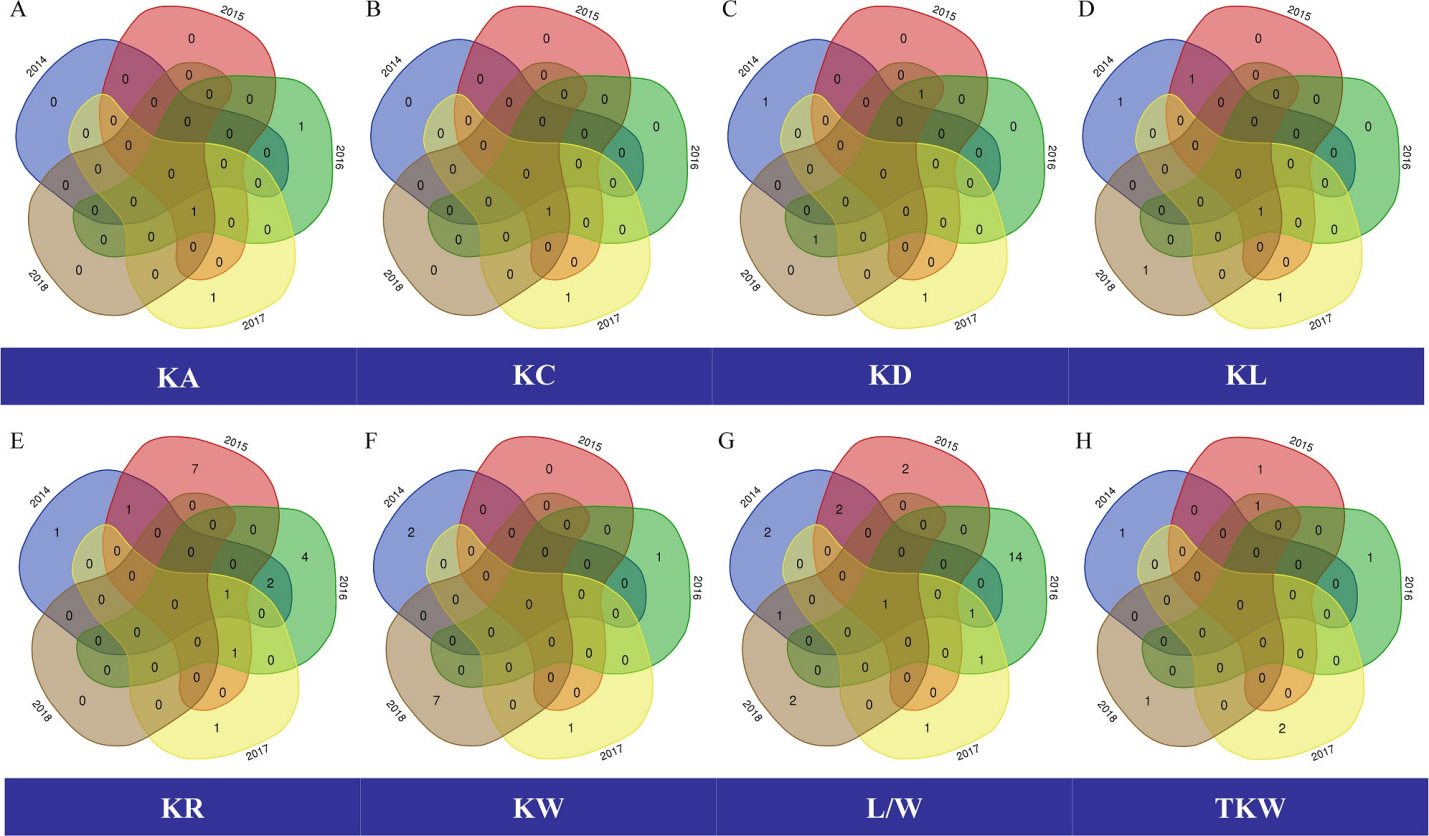
The Venn diagram of significant associations for the kernel traits in five years. **(A)** kernel area (KA), **(B)** kernel circumference (KC), **(C)** kernel diameter (KD), **(D)** kernel length (KL), **(E)** kernel roundness (KR), **(F)** kernel width (KW), **(G)** length/width ratio (L/W), **(H)** thousand kernel weight (TKW).

Only three SNP markers for KA were detected across the five years. These QTLs were located on chromosomes 5A, 5B and 6B. Out of these 3 SNPs, one repeatable SNP *BE500291_5_A_37* was detected for four consecutive years (Fig 3A), and explained 4.71–11.0% of the phenotypic variation with the highest contribution value in year 2015 (Table 2).

Only two SNP markers for KC were identified in five consecutive years. *BE500291_5_A_37* was detected from year 2015 to 2018 (Fig 3B), accounting for 5.85–12.50% of the phenotypic variance. The highest–log10 (p) value for KC was obtained from this SNP, with–log10 (p) of 4.49 in 2015 (S4 Fig). The other associated SNP marker *BF483039_7_A_Y_202* was only detected in year 2017, explaining 7.23% phenotypic variation (Table 2).

A total of 4 SNP markers for KD were obtained in five consecutive years, and located on chromosome 5A, 5B, 6B and 7B, respectively. The phenotypic variance explained (PVE) values were from 4.92% to 8.99% (Table 2). Two markers *BE500291_5_A_37* and *CD453605_6_B_427* were detected in multiple years, and the other two were year-specific markers (Fig 3C).

In total, 5 SNP markers were detected for KL, individually contributed to 4.67–12.65% of the phenotypic variance (Table 2). The highest–log10 (p) value for KL was obtained from the SNP marker *BE500291_5_A_37* in 2015 (S4 Fig). Moreover, the stable SNP *BE500291_5_A_37* was significantly associated with KL in four consecutive years, and the SNP marker *BQ168780_5_B_995* in two consecutive years were detected (Fig 3D).

Eighteen SNPs for KR were identified in five consecutive years, and were distributed more evenly on A subgenome than that on B subgenome. They individually explained 4.75–12.01% of the phenotypic variance, with *BE404912_6_B_Y_488* detected in year 2017 displaying the highest contribution value (Table 2). Five repeatable SNPs were detected in multiple years and the other 13 environment-specific SNPs were not monitored repeatedly (Fig 3E). The stable marker, *BF474023_3_A_Y_425* explained 5.01–7.80% PVE, and was observed in four consecutive years (Table 2).

Eleven SNPs for KW were obtained in five consecutive years. These SNPs were distributed on eight chromosomes, and explained 5.09–8.57% of the phenotypic variance. No SNP for KW was repeatedly detected (Table 2, Fig 3F). The SNP marker *BE499248_7_B_Y_63* on chromosome 7B was detected in year 2017 displaying the highest contribution value.

In total, 41 significant associations between L/W and SNPs were detected in five consecutive years. The SNPs markers were located in almost all chromosomes, accounting for 4.69–10.47% of the phenotypic variance. Six repeatable SNPs were mapped in multiple years. Obviously, one stable marker *BF474023_3_A_Y_425* was observed in all the five consecutive years, explaining 5.59–8.15% PVE (Table 2, Fig 3G).

Seven SNPs influencing TKW were found in five consecutive years (Fig 3H), which were relatively equally distributed on six chromosomes, and explained 4.72–9.35% of the phenotypic variance, with the highest contribution value from *BE405269_4_B_84* (Table 2).

In general, after the deletion of duplicated SNPs in Table 2, 54 unique SNP markers were found (Table 3), which distributed unevenly across almost all chromosomes except chromosomes 1A (Table 3 and S5 Fig). About half of the SNP markers were derived from four chromosomes, 2A, 6A, 4B and 6B (S5 Fig). Association analysis also showed that only 5 significant SNPs were obtained for all traits using their BLUP values (S2 Table), including *BE517914_3_A_Y_81* and *BF292264_7_A_779* associated with L/W, *BF474023_3_A_Y_425* and *BF474862_5_A_762* associated with both KR and L/W, *BE500291_5_A_37* associated with KA, KC, KD, KL and TKW (S2 Table). Combining all significant associations identified from annual data and BLUP data together, 19 repeatable associations, each of which was detected in two or more environments, were identified from different evaluated traits (S3 Table). For example, BF474023_3_A_Y_425 associated with L/W was observed in all environments, and other SNPs were detected in two to five environments.

**Table 3:** Candidate SNP loci identified in this study overlapping the regions of previously known QTLs in durum genome.

Marker^a^	Position of marker^b^			Known QTL of kernel-relative trait^c^				
	Chr	Start	End	Name	Position	Physical distance	Trait	Reference
BG263233_1_A_Y_836	chr1B	600676184	600676425	QTL1735_1B	chr1B:33986133–594220377	560234244	kernel weight	Peng et al. [38]
BE405604_2_A_Y_353	chr2A	461924254	461924495	QTL0677_TKW	chr2A:194985905–605115332	410129427	kernel weight	Mangini et al. [39]
BE446480_2_A_N_24	chr2A	106406444	106406583	n.d				
BE488358_2_B_N_620*	chr2A	767209359	767209599	QTL1745_2A	chr2A:761215833–775446234	14230401	kernel weight	Peng et al. [38]
				QTL1518_2A	chr2A:753056610–775446234	22389624	kernel weight	Maccaferri et al. [40]
				QTL0680_TKW	chr2A:727267935–748477405	21209470	kernel weight	Mangini et al. [39]
BG605368_2_A_156	chr2A	706486440	706486681	QTL1718_2A	chr2A:701400169–731617683	30217514	kernel weight	Peleg et al. [41]
BQ168780_5_B_995	chr2A	57409746	57409881	n.d				
BE498892_2_A_208	chr2A	757559658	757559898	QTL1518_2A	chr2A:753056610–775446234	22389624	kernel weight	Maccaferri et al. [40]
BE403597_2_B_Y_552	chr2B	489898967	489899208	QTL1130_2B	chr2B:196546476–537614490	341068014	kernel weight	Faris et al. [42]
BE404332_2_B_29	chr2B	346924954	346925043	QTL1130_2B	chr2B:196546476–537614490	341068014	kernel weight	Faris et al. [42]
BE406351_2_B_Y_100	chr2B	493058796	493059015	QTL1130_2B	chr2B:196546476–537614490	341068014	kernel weight	Faris et al. [42]
BE517872_2_A_N_504	chr2B	228825939	228826173	QTL1130_2B	chr2B:196546476–537614490	341068014	kernel weight	Faris et al. [42]
BE517914_3_A_Y_81	chr3A	595302050	595302247	QTL0968_3A	chr3A:543536695–565652591	22115896	kernel weight	Blanco et al. [43]
BE425919_3_A_592	chr3A	470072941	470073107	n.d				
BF474023_3_A_Y_425	chr3A	425204392	425204151	n.d				
CD452413_3_B_Y_189	chr3B	676319519	676319760	n.d				
BE445508_3_B_Y_209	chr3B	357213589	357213830	QTL1399_3B	chr3B:195223768–456085142	260861374	kernel weight	Graziani et al. [44]
BE446087_3_B_Y_750	chr3B	125801022	125801223	n.d				
BE495175_3_B_Y_443*	chr3B	752255163	752255404	QTL1134_3B	chr3B:736501649–772286011	35784362	kernel weight	Faris et al. [42]
				QTL0686_TKW	chr3B:742628705–774081325	31452620	kernel weight	Mangini et al. [39]
BE352626_4_A_Y_110	chr4A	102124871	102125100	n.d				
BE425301_4_A_Y_160	chr4A	36377171	36377412	n.d				
BE495116_4_A_Y_239	chr4A	179488191	179488432	QTL0692_TKW	chr4A:103274341–576057502	472783161	kernel weight	Mangini et al. [39]
BE591739_4_A_Y_131	chr4A	265646	265887	n.d				
BE404377_4_B_Y_333*	chr4B	185966723	185966964	QTL1725_4B	chr4B:35826374–526354820	490528446	kernel weight	Peleg et al. [41]
				QTL0905_TKW	chr4B:35826374–449274630	413448256	kernel weight	Soriano et al. [45]
				QTL1676_4B	chr4B:180052042–504276765	324224723	kernel weight	Patil et al. [46]
BE442666_4_B_Y_327*	chr4B	25926747	25926988	QTL0902_TKW	chr4B:27158917–180051943	152893026	kernel weight	Soriano et al. [45]
				QTL2013_4B	chr4B:22216806–32837740	10620934	kernel weight	Russo et al. [47]
BE404977_4_B_Y_227*	chr4B	26664293	26664534	QTL2013_4B	chr4B:22216806–32837740	10620934	kernel weight	Russo et al. [47]
				QTL0902_TKW	chr4B:27158917–180051943	152893026	kernel weight	Soriano et al. [45]
BE405269_4_B_84	chr4B	643901320	643901523	QTL1406_4B	chr4B:624077361–653894380	29817019	kernel weight	Graziani et al. [44]
BE443253_4_B_Y_414*	chr4B	396507651	396507832	QTL1725_4B	chr4B:35826374–526354820	490528446	kernel weight	Peleg et al. [41]
				QTL0905_TKW	chr4B:35826374–449274630	413448256	kernel weight	Soriano et al. [45]
				QTL1676_4B	chr4B:180052042–504276765	324224723	kernel weight	Patil et al. [46]
BF482356_4_B_Y_504*	chr4B	338755473	338755655	QTL1725_4B	chr4B:35826374–526354820	490528446	kernel weight	Peleg et al. [41]
				QTL0905_TKW	chr4B:35826374–449274630	413448256	kernel weight	Soriano et al. [45]
				QTL1676_4B	chr4B:180052042–504276765	324224723	kernel weight	Patil et al. [46]
BG604507_4_B_383*	chr4B	120944366	120944607	QTL1725_4B	chr4B:35826374–526354820	490528446	kernel weight	Peleg et al. [41]
				QTL0905_TKW	chr4B:35826374–449274630	413448256	kernel weight	Soriano et al. [45]
				QTL0902_TKW	chr4B:27158917–180051943	152893026	kernel weight	Soriano et al. [45]
				QTL1547_4B	chr4B:87540945–180051943	92510998	kernel weight	Maccaferri et al. [40]
BE500291_5_A_37	chr5A	149303640	149303797	QTL0201_TKW	chr5A:43811436–321137020	277325584	kernel weight	Kidane et al. [48]
BE403211_5_A_Y_601	chr5A	584269900	584270062	n.d				
BG274985_5_A_Y_267	chr5A	312216001	312216242	QTL0201_TKW	chr5A:43811436–321137020	277325584	kernel weight	Kidane et al. [48]
CD452967_5_B_Y_229*	chr5B	121165117	121165358	QTL0204_TKW	chr5B:46147603–132851290	86703687	kernel weight	Kidane et al. [48]
				QTL0205_TKW	chr5B:57572552–370924682	313352130	kernel weight	Kidane et al. [48]
				QTL2045_5B	chr5B:68504586–497044688	428540102	kernel weight	Thanh et al. [49]
				QTL2044_5B	chr5B:68504586–467639351	399134765	kernel weight	Thanh et al. [49]
BE496986_6_A_110	chr6A	536591248	536591477	n.d				
BE498763_6_A_Y_318*	chr6A	459213165	459213406	QTL1416_6A	chr6A:129338267–480593476	351255209	kernel weight	Graziani et al. [44]
				QTL0719_TKW	chr6A:72138718–493284322	421145604	kernel weight	Mangini et al. [39]
BE636872_6_A_119	chr6A	609352251	609352013	QTL1361_6A	chr6A:598732579–608245286	9512707	kernel weight	Golabadi et al. [50]
BF483091_6_A_357	chr6A	598092326	598092567	QTL1361_6A	chr6A:598732579–608245286	9512707	kernel weight	Golabadi et al. [50]
BG262421_6_A_87*	chr6A	83401625	83401831	QTL0719_TKW	chr6A:72138718–493284322	421145604	kernel weight	Mangini et al. [39]
				QTL1729_6A	chr6A:72138718–129338366	57199648	kernel weight	Peleg et al. [41]
BE404912_6_B_Y_488	chr6B	352351449	352351690	QTL1965_6B	chr6B:152609610–455751843	303142233	kernel weight	Roncallo et al. [51]
				QTL1966_6B	chr6B:204758239–525347563	320589324	kernel weight	Roncallo et al. [51]
BQ161779_6_B_Y_185*	chr6B	551413872	551414113	QTL2070_6B	chr6B:453208656–554734719	101526063	kernel weight	Tzarfati et al. [52]
				QTL1121_6B	chr6B:551574028–608165141	56591113	kernel weight	Elouafi et al. [53]
BE445667_6_B_Y_285	chr6B	251174736	251174977	QTL1965_6B	chr6B:152609610–455751843	303142233	kernel weight	Roncallo et al. [51]
				QTL1966_6B	chr6B:204758239–525347563	320589324	kernel weight	Roncallo et al. [51]
BE604119_6_B_733	chr6B	294863898	294864064	QTL1965_6B	chr6B:152609610–455751843	303142233	kernel weight	Roncallo et al. [51]
				QTL1966_6B	chr6B:204758239–525347563	320589324	kernel weight	Roncallo et al. [51]
BF291774_6_B_519*	chr6B	130985014	130985221	QTL1417_6B	chr6B:64021852–118795530	54773678	kernel weight	Graziani et al. [44]
				QTL1730_6B	chr6B:74746310–151031346	76285036	kernel weight	Peleg et al. [41]
				QTL1120_6B	chr6B:64021852–110377320	46355468	kernel weight	Elouafi et al. [53]
CD453605_6_B_427	chr6B	10805766	10806007	QTL0920_TKW	chr6B:14426556–39461288	25034732	kernel weight	Soriano et al. [45]
BE497375_7_A_Y_191	chr7A	479765892	479766133	n.d				
BE499652_7_A_Y_391	chr7A	157545898	157546139	QTL1684_7A	chr7A:106152757–131796856	25644099	kernel weight	Patil et al. [46]
BE637838_7_A_Y_208	chr7A	689303442	689303683	QTL0731_TKW	chr7A:694638997–717853890	23214893	kernel weight	Mangini et al. [39]
BF292264_7_A_779	chr7A	18762196	18762437	QTL0160_TKW	chr7A:23744953–29299532	5554579	kernel weight	Giraldo et al. [54]
BF292193_7_B_N_78	chr7B	576058434	576058629	QTL0737_TKW	chr7B:496126667–578606740	82480073	kernel weight	Mangini et al. [39]
BE443010_7_B_354*	chr7B	503240339	503240580	QTL0737_TKW	chr7B:496126667–578606740	82480073	kernel weight	Mangini et al. [39]
				QTL1982_7B	chr7B:411627186–496126441	84499255	kernel weight	Roncallo et al. [51]
				QTL0979_7B	chr7B:459321833–517442227	58120394	kernel weight	Blanco et al. [43]
BE499248_7_B_Y_63*	chr7B	26282735	26282917	QTL1981_7B	chr7B:856388–45454444	44598056	kernel weight	Roncallo et al. [51]
				QTL1733_7B	chr7B:2357279–7257942	4900663	kernel weight	Peleg et al. [41]
				QTL1425_7B	chr7B:6464988–22197787	15732799	kernel weight	Graziani et al. [44]
BF483039_7_A_Y_202	chrUn	153138960	153139201	n/a				
BM140538_2_B_133	chrUn	34717974	34718218	n/a				
BF474862_5_A_762	chrUn	68232437	68232678	n/a				

* represents the SNP markers overlapped with two or more known QTLs.

^a^ Candidate SNP markers identified in this study.

^b^ The position of marker in durum genome.

^c^ QTLs for kernel-relative traits reported in previous studies.

Chr, chromosome. n.d, not denoted; n/a, not applicable.

Based on the association study using BLUP values across the five consecutive years, the number of SNP markers associated with L/W was relatively higher than other kernel traits, most of which only have one marker (S2 Table). Thus, haplotype study was carried out on the L/W trait with the number of favored alleles. Seven haplotypes were identified across four significant SNPs (S4 Table). Among lines having 1 to 2 favorable alleles, the values of L/W were relatively higher, while with increasing numbers of favorable alleles the values were decreased. Accordingly, it was shown negative correlation between the L/W and the number of favorable alleles (R^2^ = 0.527) using linear regression analysis (S3D Fig).

### Combination Analysis of Loci Identified here with Previously Known QTL

In previous studies of kernel traits in durum, only kernel weight-related QTLs have been identified using traditional linkage mapping and genome-wide association mapping [38–54]. After searching QTL identified here with previously reported QTL in durum wheat genome, most of the SNPs identified from kernel-related traits in this study were close to or overlapped with the positions of kernel weight-related QTL reported in previous studies. The loci of twelve SNPs identified in this study were not detected previously (Table 3). In addition, each of the eight SNP markers *BE488358_2_B_N_620*, *BE404377_4_B_Y_333*, *BE443253_4_B_Y_414*, *BF482356_4_B_Y_504*, *BG604507_4_B_383*, *BF291774_6_B_519*, *BE443010_7_B_354* and *BE499248_7_B_Y_63*, was overlapped with more than three known QTLs detected for kernel-weight traits in previous reports (Table 3). Interestingly, half of them were located on chromosome 4B.

Moreover, all four SNPs *BE403597_2_B_Y_552*, *BE404332_2_B_29*, *BE406351_2_B_Y_100* and *BE517872_2_A_N_504* from chromosome 2B were located on the same known QTL of *QTL1130_2B* with physical distance of 341 Mb between chr2B-196546476 and chr2B-537614490 (Table 3). Two adjacent SNPs, *BE404977_4_B_Y_227* and *BE442666_4_B_Y_327*, were found in the same QTL region of *QTL2013_4B* on chromosome 4B. It also has two SNP markers close to both sides of the same QTL region of *QTL1361_6A* with *BE636872_6_A*_119 and BF483091_6_A_357 on each side. Meanwhile, the physical areas for these two QTL were relative narrow, only about 10 Mb of chromosome regions for both *QTL2013_4B* and *QTL1361_6A* (Table 3). In addition, the two QTLs, *QTL0160_TKW* flanked by *BF292264_7_A_779* and *QTL1733_7B* overlapped with *BE499248_7_B_Y_63*, have the minimum physical interval of about 5 Mb (Table 3).

### Identification of candidate genes

Since the resolution was very low and LD were significantly large in this study, it would be rather difficult to define candidate genes. As the SNP markers used in this study were developed from the EST database, so these SNPs were actually expressed genes in wheat. Thus, the EST sequences related to candidate SNP markers were analyzed by using BLAST at the NCBI for gene function prediction. As the result, a total of 54 candidate genes supposed to be important for kernel traits were annotated from the significantly associated markers in this study (S5 Table). The candidate genes were divided into several categories, most of them encoded metabolism related enzymes, and some of them involved in kernel development. A comparison of SNPs detection by using five-years BLUP values also indicated the most consistent association for kernel traits was the same SNP of *BE500291_5_A_37* (S3 Table). The sequence of this stable marker was derived from wheat pre-anthesis spike cDNA library, whose functional annotation was best matched with 1-acyl-sn-glycerol-3-phosphate acyltransferase (PLS1). Thus, PLS1 gene might play a core role in grain development in durum wheat. Another important SNP locus, *BF474023_3_A_Y_425* located on chromosome 3A was simultaneously detected in all of the five environments for L/W (Table 2), whose functional annotation is abscisic acid insensitive like1 protein (ABIL1) (S5 Table). It can be considered that PLS1 and ABIL1 are two of the most important genes that determine grain architecture in durum.

### Relationship between climatic variables and kernel traits

**Fig 4:**
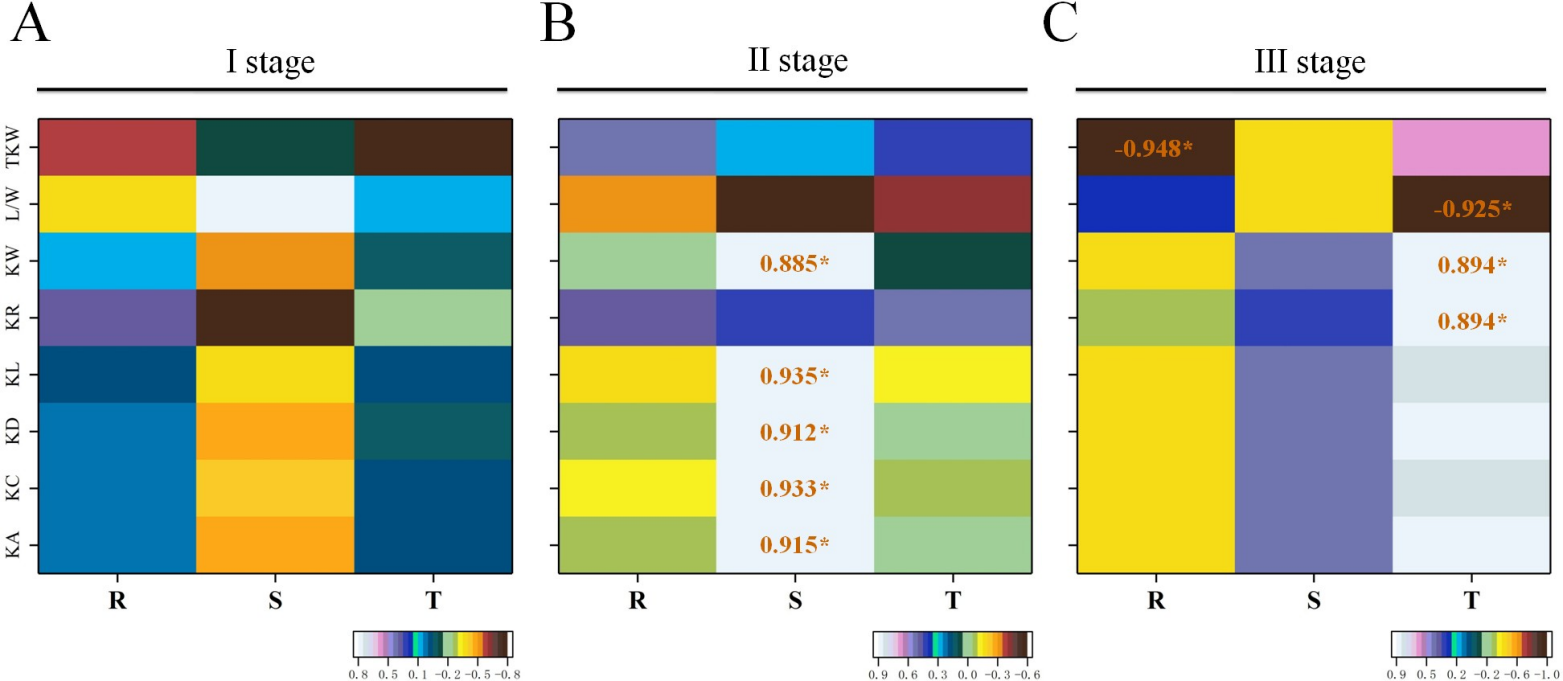
Correlation coefficients of kernel phenotypes with climate factors based on mean values. **(A)** stage I represented the regreening period of in February. **(B)** stage II corresponded with the jointing stage about in March. **(C)** stage III was the combination period from heading to ripening (April-June period). *T* temperature, *S* sunlight, *R* rainfall precipitation. *Significant at P  < 0.05; **significant at P  < 0.01.

Climate variability is one of the most important factors for crop production. In order to evaluate the potential impact of climatic factors on kernel growth, a preliminary analysis has been performed on their association. We collected five years of meteorological information from weather station in China's central Hubei province (S6 Table). Correlation analysis showed that there was no significant correlation between climatic variables and kernel traits at stage I (Fig 4A). However, the significant and positive correlations were found between temperature and five kernel traits except KR, and L/W and TKW at stage II (Fig 4B). Furthermore, the correlation analysis indicated that significant and positive correlations were presented between temperature and both KR and KW traits, while significant and negative correlation between temperature and L/W at stage III (Fig 4C). In addition, the average of rainfall precipitation was negatively correlated with almost all of the kernel traits, and exhibited significant and negative correlations with TKW at stage III (Fig 4C).

## Discussion

GWAS is the most popular approach for dissecting the genetic constitution of the heritable complex traits [55]. So far, it has been successfully used in the exploration of candidate genes in durum [56]. However, few genes/QTLs associated with kernel traits have been identified in durum wheat through the association mapping approach. In this study, we intended to reveal the genetic architecture of kernel characters in a panel of 150 durum lines collected from 46 countries and regions. A lot of SNPs associated with kernel-related traits were identified. Our results provide a useful resource for further functional studies to understand the molecular mechanism of the regulation involved in grain development.

### The stable SNPs for controlling a trait or different traits

The effect of association analysis could be impacted by genetic and environmental factors [57]. In order to increase the reliability of SNPs identified, a total of five year data were used to identify associations for kernel traits in our study. A considerable number of SNP markers were detected in more than two environments and exhibited obvious environmental stability (Table 3 and S3 Table). Therefore, the more consistency of obtained a SNP for a kernel trait across different environments implied, the more importance of itself in kernel development. For instance, *BF474023_3_A_Y_425* was repeatedly detected for L/W in all environments. Thus, this locus may play an important potential role in kernel development. However, all SNP markers identified for KW in wheat had poor stability, which were detected only in a single year. The results implied that kernel width might be controlled and modified by more minor effect genes. The multiple effects of a single gene on different phenotypic traits are the phenomenon of gene pleiotropy [58]. Many candidate genes tagged by SNP markers may control multiple kernel-related traits in this study. Observably, sixteen important pleiotropic loci were further identified by overlapping analysis (S7 Table). In particular, *BE500291_5_A_37*, one stable SNP for KA, KC, KD, KL, L/W and TKW, was repeatedly detected in two or more environments for each associated trait with less environmental interactions (Table 3). Therefore, the candidate gene marked by *BE500291_5_A_37* may be a critical regulator for kernel development.

### Molecular mechanisms underlying kernel-related traits

It is difficult to define candidate genes as the low resolution and large LD in this study. Nevertheless, the SNP markers used in this study was developed from the EST database, these ESTs might be candidate genes. As this study shown, the stable SNP marker *BE500291_5_A_37*, concurrently associated with six of kernel-related traits, which provided a candidate for further studying its function on grain development. Moreover, some other genes tagged by the EST-derived SNP markers may play roles in kernel development of durum wheat. The EST of *BF482356_4_B_Y_504* was shown very high homology with the ubiquitin carboxyl-terminal hydrolase 12 (S5 Table). The E3 Ubiquitin ligase OsGW2 is associated with rice grain development by influencing kernel width and weight. Its homologue gene, located on the homologous group 6 chromosomes in wheat [59], was also identified and considered as a candidate gene related to grain weight and width [60]. Thus, a new ubiquitin-mediated pathway contributed to kernel development in durum might be controlled by the gene marked by *BF482356_4_B_Y_504* on chromosome 4B. Because of the role of auxin in regulating grain size, plant productivity could be improved by altering auxin transport and distribution [61]. Consequently, the low expression of *TaTGW6* was associated with low auxin content that was considered to be the main influence factor for grain development of wheat [62]. In this study, the SNP marker *BE497375_7_A_Y_191*, significantly associated with KW (Table 3), was found to be very high homology with auxin-responsive protein IAA21 (S5 Table). Therefore, we speculated that the contribution of *BE497375_7_A_Y_191* to KW might be attributed to the role of IAA signaling pathway. Abscisic acid-response genes have effects on accumulation of storage proteins and participate in seed development, such as in Arabidopsis and soybean [63, 64]. The EST of *BF474023_3_A_Y_425* has very high homology with an ABA insensitive protein encoded by *ABIL1* (Abscisic acid insensitive like 1). Therefore, the responsive gene involved in ABA signaling pathway might be correlated with grain development of durum.

### Syntenic regions of candidate genes in 5A chromosome

In the present study, many SNP markers were identified for kernel-related traits in different years in durum wheat. In which, a stable and multi-traits associated locus *BE500291_5_A_37* was mapped on chromosome 5A (Table 3 and S5 Fig), which can be further explored for discovering candidate genes and for function analysis across traits and environments. Integrated with our published studies [36, 65, 66], we further picked out all of those significant SNPs which we had previously found in chromosome 5A. In total, 23 unique significant SNPs were associated with 41 evaluated traits at different developmental stages of vegetative and reproductive growth in durum (S8 Table). According to the durum genome sequence information, several of them were clustered on 5A region with a short physical distance of 31 Mb (S6 Fig), implying that this region might be SNP hotspots. Meanwhile, co-localizing SNPs were identified among seedling traits, canopy leaf traits, agronomic traits, and kernel traits. Especially, a SNP *BE443538_5_A_1436* associated with 19 traits was deemed to be a super pleiotropic marker that was highly related with the growth and development of durum. Therefore, the candidate genes close to *BE443538_5_A_1436* might affect multi-phenotypes in durum. Therefore, this region from 129–160 Mb encompassed by six SNP markers on chromosome 5A was supposed to be the crucial candidate region for gene discovery in our future work.

### The impact of climate variability on kernel traits

In this study, phenotypes of some kernel traits seemed to be affected by environments, presenting different trends in different years, the values in the years from 2014 to 2016 were all significant lower than those from 2017 to 2018 (S1 Fig). Previous research has demonstrated that the final grain yield is controlled by a network of genes and environment factors [67], such as temperature, sunlight, and rainfall precipitation. Especially, temperature was the major governing factor during crop growth period [68]. The variation in average growing-season temperatures of ±2°C can cause reduction in grain production up to 50% for wheat in Australia [69]. It was proposed that global wheat production will change by −2.3% to 7.0% under the 1.5°C warming and −2.4% to 10.5% under the 2.0°C warming [70]. There were few reports about the relationship between grain size and climate in wheat. Previous study showed that the low average temperature in March and April greatly increased grain number per spike, and the longer sunshine duration could increase grain weight in north China [71]. Similarly, the longer sunshine duration at II stage could ultimately increase KA, KC, KD, KL and KW of grain in durum. This result suggested that the role of sunshine duration is quite important in durum growth at jointing stage (Fig 4B). Moreover, temperature showed significant correlations with both KR and KW in the period from heading to ripening, but there were no significant correlations between KL and climate factors in this stage (Fig 4C). Therefore, KL had more climate stability than other evaluated traits. Furthermore, the average precipitation was negatively correlated with almost all of the kernel traits, as well as exhibited significant negatively correlation with TKW (Fig 4C). This indicated the larger amount of precipitation, the less kernel dimension and especially the kernel weight. The research about the adaptation of wheat to areas of Europe indicated that the hotter and drier climate was concerned with quicker maturation, but resulting in lower yields [72]. Similarly, our study implied that colder and moister climate might particularly contribute to lower grain quality of wheat in Middle-lower Yangtze River area in China. Conclusively, the large grain dimension and high grain weight needed a longer sunshine duration, a moderate temperature and certain amount of precipitation at different developmental stages in durum.

## Conclusions

To increase yield is still the main goal in common wheat breeding until now. One of the important facets to achieve this goal is to explore novel genetic resources to discover genes that affect grain yield. In this study, association analysis for kernel characters in a natural population of durum wheat was conducted using genome-wide of EST-derived SNP markers. Consequently, 54 significantly unique SNP markers were identified from 109 marker-trait association pairs. Especially, the stable SNP *BE500291_5_A_37* was repeatedly detected in two or more environments for each associated trait. The candidate loci identified for controlling kernel traits in durum will provide candidates for studying the genetic architecture of grain quality in common wheat.

## Supporting information

S1 Fig Boxplot of the phenotypic data of eight evaluated kernel traits for the durum wheat natural population in five years. Analysis of variance (ANOVA) was applied to examine the difference of traits among different years. Different numbers indicate statically significant difference at P ≤ 0.05. Phenotypic differences obsered for each trait under five consecutive years of 2014–2018, respectively. (A) KA; (B) KC; (C) KD; (D) KL; (E) KR; (F) KW; (G) L/W; (H) TKW.
(TIF)

S2 Fig Comparison of kernel-related traits between landraces and cultivars. Analysis of variance (ANOVA) was applied to examine the difference of traits between landraces and cultivars. There was no significant difference between the two groups (P values > 0.05).
(TIF)

S3 Fig Associations for kernel-related traits and haplotype study for L/W. **(A)** SNP numbers for every kernel-related trait in different years. **(B)** The range of associated R^2^-values (variation explained by SNP markers) distributed for each kernel trait detected under five years of 2014–2018. **(C)** The distribution of R^2^-values for each kernel trait evaluated by using five-years best linear unbiased prediction (BLUP) values. **(D)** Linear regressions between number of favorable alleles and mean phenotypic effect on L/W.
(TIF)

S4 Fig Manhattan plots for kernel traits and summary of several important SNP markers identified from association analysis. (A) Manhattan plots of P values indicating SNP markers associated with KC in 2015. (B) Manhattan plots of P values indicating SNP markers associated with KL in 2015. The horizontal line indicated P = 0.01 thresholds for significant associations.
(TIF)

S5 Fig Chromosomal locations of significant SNPs for kernel-related traits identified in this study. Positions of significant markers projected to the durum wheat genome (*Triticum turgidum* Durum Wheat Svevo, RefSeq Rel. 1.0).
(TIF)

S6 Fig A physical region of the associated SNP markers on 5A chromosome segment from 129 to 160 Mb. (TIF)

S1 Table Number of SNP marker-trait associations for the observed traits in different years. (XLS)

S2 Table Significant association pairs between SNP markers and kernel traits detected by using five-years BLUP values. (XLS)

S3 Table Significant association pairs between SNP markers and kernel traits detected at least in two environments. (XLS)

S4 Table Haplotypes analysis using four SNPs and their phenotypic effects. Marked base representing favorable alleles.
(XLS)

S5 Table The putative functions of candidate genes for each significant SNPs analyzed by BLAST alignment using their EST sequences. (XLS)

S6 Table The average values of rainfall precipitation, temperature and sunlight during three important growth stages in Hubei from 2014 to 2018. (XLS)

S7 Table SNP markers associated with multiple kernel traits in durum wheat. (XLS)

S8 Table SNP markers on 5A chromosome associated with multiple traits for different developmental stages of vegetative and reproductive growth in durum wheat. (XLS)
