# Supplementary figures and images for: SNP-based association study of kernel architecture in a worldwide collection of durum wheat germplasm
Source: PLoS One. 2020 Feb 14;15(2):e0229159. doi: 10.1371/journal.pone.0229159 (PMC7021289; doi:10.1371/journal.pone.0229159)

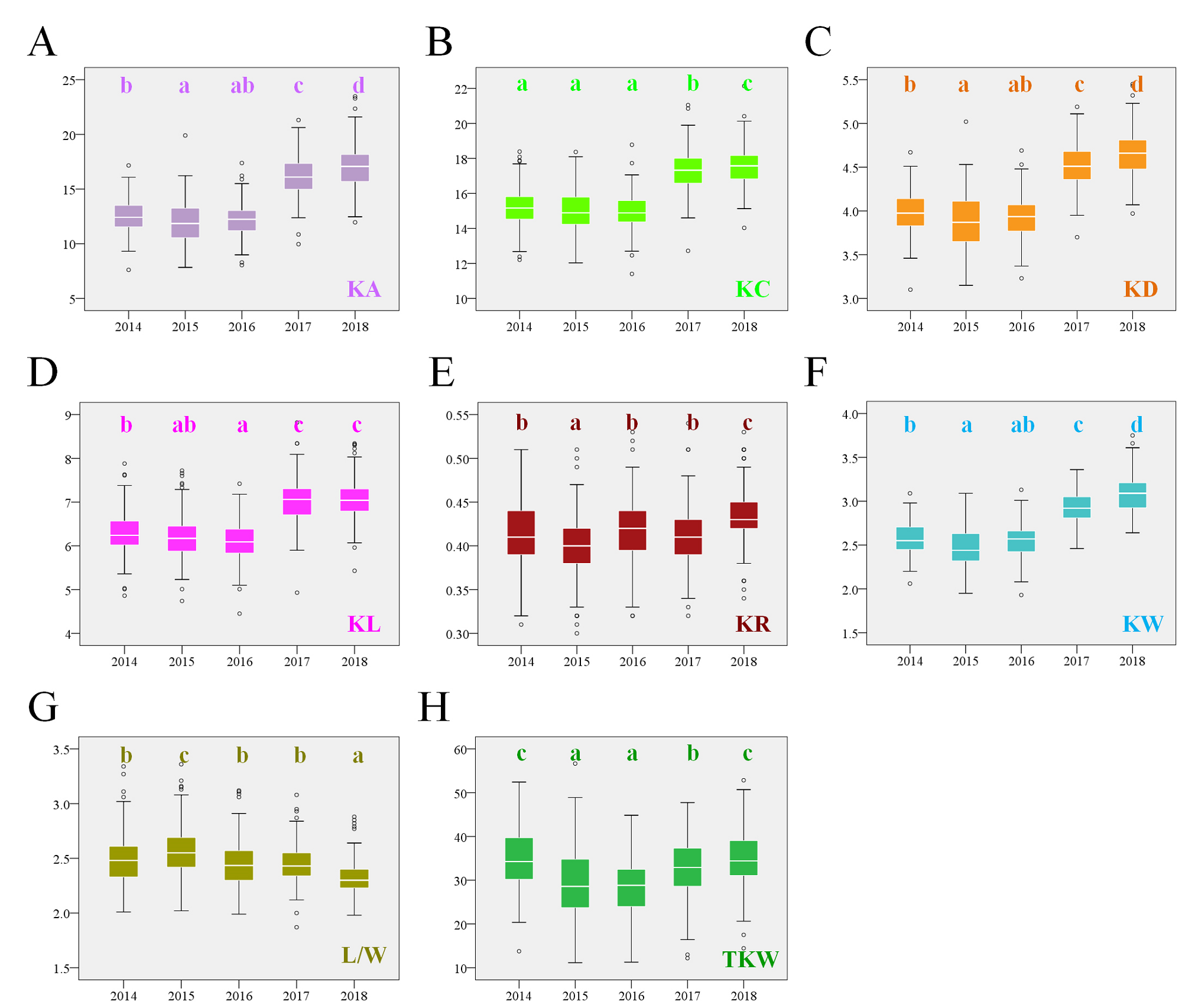

Supplement: S1 Fig — Analysis of variance (ANOVA) was applied to examine the difference of traits among different years. Different numbers indicate statically significant difference at P ≤ 0.05. Phenotypic differences obsered for each trait under five consecutive years of 2014–2018, respectively. (A) KA; (B) KC; (C) KD; (D) KL; (E) KR; (F) KW; (G) L/W; (H) TKW. (TIF) [file pone.0229159.s001.tif]

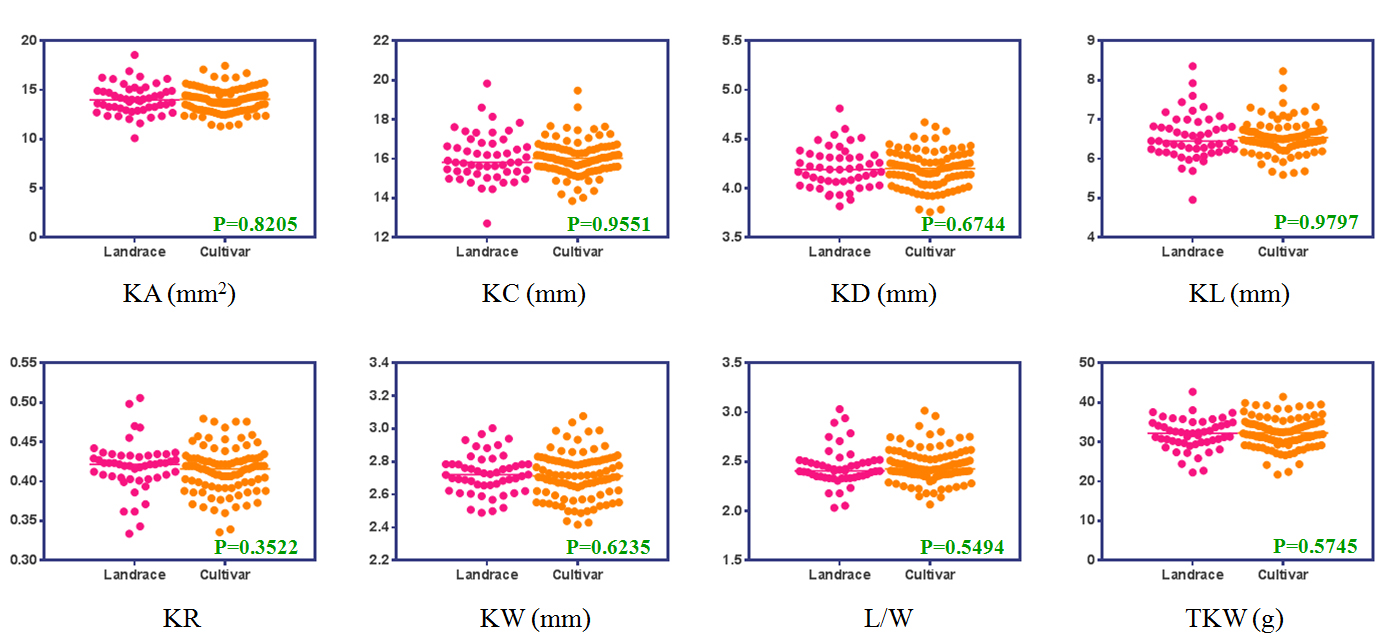

Supplement: S2 Fig — Analysis of variance (ANOVA) was applied to examine the difference of traits between landraces and cultivars. There was no significant difference between the two groups (P values > 0.05). (TIF) [file pone.0229159.s002.tif]

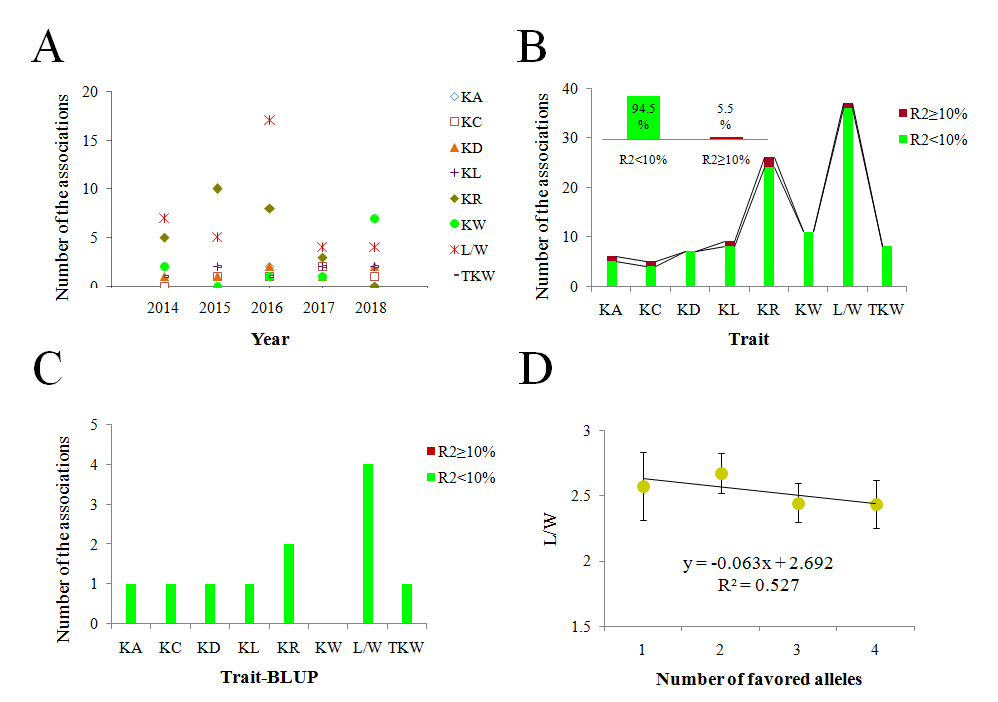

Supplement: S3 Fig — (A) SNP numbers for every kernel-related trait in different years. (B) The range of associated R2-values (variation explained by SNP markers) distributed for each kernel trait detected under five years of 2014–2018. (C) The distribution of R2-values for each kernel trait evaluated by using five-years best linear unbiased prediction (BLUP) values. (D) Linear regressions between number of favorable alleles and mean phenotypic effect on L/W. (TIF) [file pone.0229159.s003.tif]

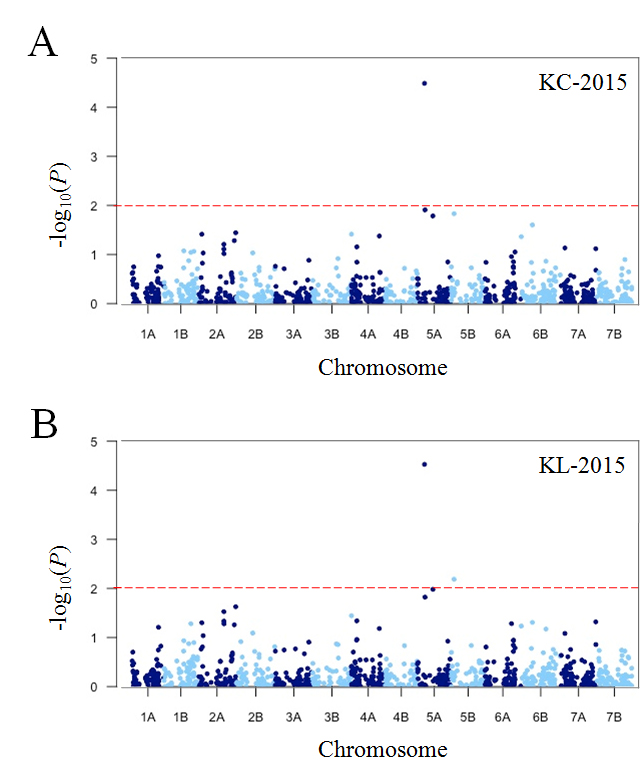

Supplement: S4 Fig — (A) Manhattan plots of P values indicating SNP markers associated with KC in 2015. (B) Manhattan plots of P values indicating SNP markers associated with KL in 2015. The horizontal line indicated P = 0.01 thresholds for significant associations. (TIF) [file pone.0229159.s004.tif]

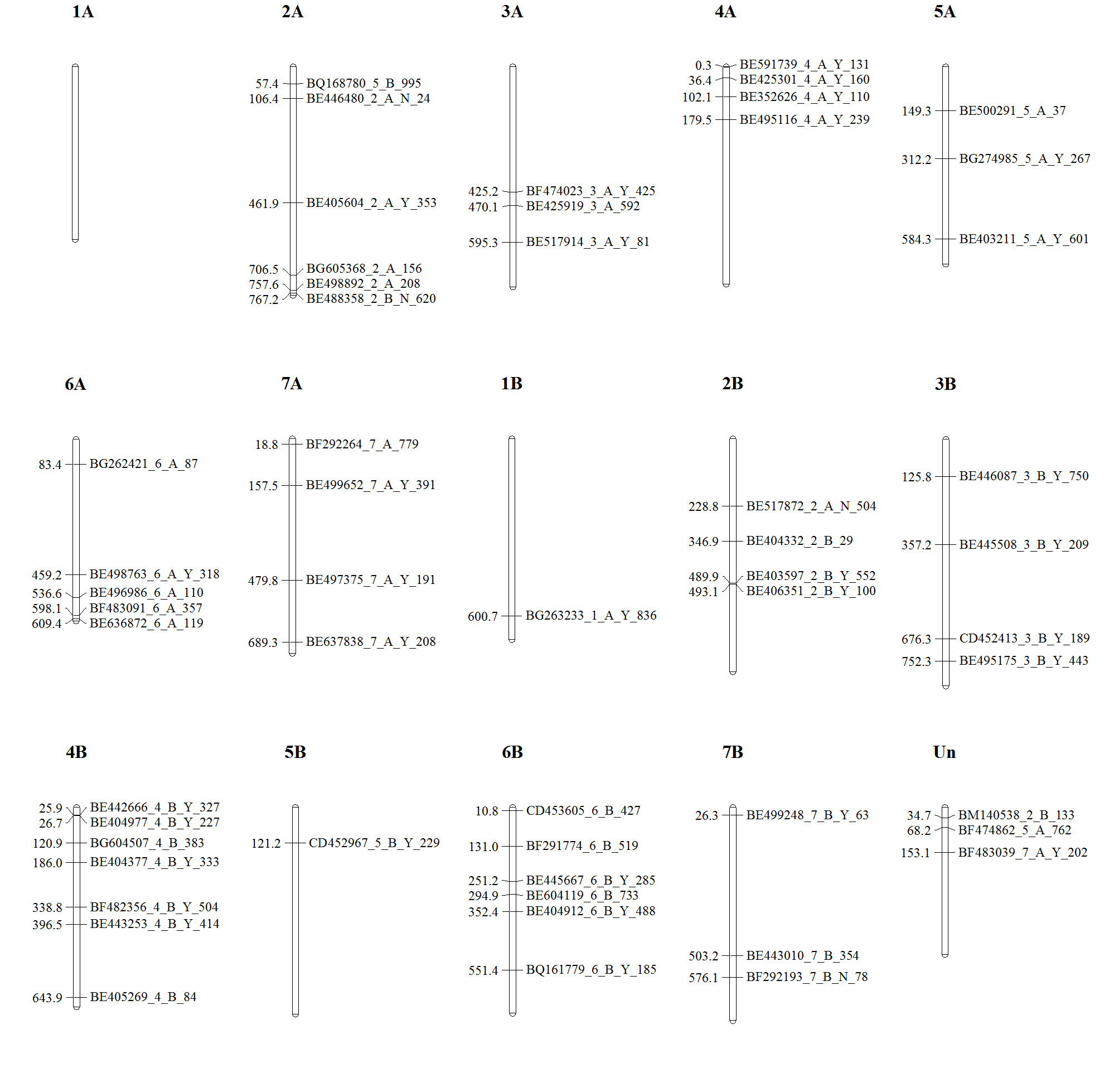

Supplement: S5 Fig — Positions of significant markers projected to the durum wheat genome (Triticum turgidum Durum Wheat Svevo, RefSeq Rel. 1.0). (TIF) [file pone.0229159.s005.tif]

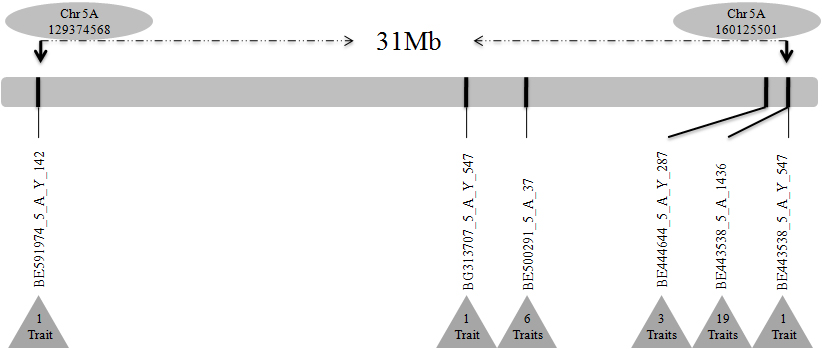

Supplement: S6 Fig — (TIF) [file pone.0229159.s006.tif]
